# Impact of One-Year Methadone Maintenance Treatment in Heroin Users in Jiangsu Province, China

**DOI:** 10.4137/sart.s2914

**Published:** 2009-09-23

**Authors:** Guohong Chen, Takeo Fujiwara

**Affiliations:** 1 Center for Disease Prevention and Control, Jiangsu Province, China 210009.; 2 Master of Public Health course, National Institute of Public Health, Saitama, Japan 351-0197.; 3 Department of Health Promotion, National Institute of Public Health, Saitama, Japan 351-0197. Email: jscares@hotmail.com

**Keywords:** impact, methadone maintenance treatment, HIV infection, heroin users

## Abstract

**Context:**

Although the effectiveness of methadone maintenance treatment (MMT) is well-established in many countries, it is a relatively new therapy for heroin users in China. Jiangsu Province, a relatively wealthy province, set up 4 MMT clinics in February 2006. No previous studies have evaluated the impact of MMT in a wealthy Chinese province.

**Objective:**

The aim of this study is to evaluate the impact of a 1-year MMT among heroin users in Jiangsu Province. We investigated the impact of the treatment by examining the following outcomes: 1) reduction of heroin use, 2) increase of appropriate sexual intercourse, 3) reduction of antisocial behavior, 4) increase of better social and family relationships, and 5) HIV prevalence among heroin users in MMT clinics.

**Design and Setting:**

Repeated cross-sectional surveys were conducted before and after heroin users in Jiangsu Province received at least 1-year of treatment in the MMT clinics. A questionnaire survey was implemented for those who agreed to participate from March to April 2006, before the initiation of MMT (N = 554). The second survey was from August to September 2007 and was administered to those who received MMT for more than 1 year (N = 804). One hundred and ninety-six patients who were investigated in both surveys were included in a longitudinal study to evaluate the factors attributable to behavior change.

**Results:**

MMT helped in reducing the percentage of heroin injection and also improved social and familial relationships. Antisocial behavior, including theft, prostitution, and dealing in heroin, decreased after 1-year treatment in the MMT clinics. However, the percentage of patients using condoms was not statistically significant. No case was found to be HIV-positive among those who received more than 1 year MMT. In the longitudinal study of 196 patients who participated in both surveys, no specific demographic variables were found to be associated with heroin use, anti-social behaviors after 1-year MMT.

**Conclusions:**

MMT was thought to reduce heroin use, antisocial behaviors and HIV prevalence, and increased appropriate sexual intercourse behaviors and better social and family relationships among heroin users in a wealthy province in China, which was true regardless of gender, age, marital status, or working status.

## Introduction

Fifty thousand newly discovered HIV-positive cases were reported in China in 2007. HIV transmission through shared syringes accounted for 42% of all these new cases; this was followed by homosexual transmission and mother-to-child transmission.

Methadone maintenance treatment (MMT) is a replacement treatment for heroin addiction. It is used worldwide and this treatment is legal, convenient, safe, and effective.^1^–^5^ There were 260,000 registered patients who took methadone daily in 2008 throughout the United States.^6^

In China, MMT is a relatively new therapy that was launched in 2004 and is being implemented on a large scale in order to prevent HIV transmission among heroin users. By the end of 2006, a total of 37,345 patients had been treated in 320 clinics across the entire nation.^7^ Pang et al conducted a repeated cross-sectional study, one at 6 months and the other after 1 year, to assess the effectiveness of MMT in the first 8 clinics in 5 provinces, i.e. Sichuan, Yunnan, Guizhou, Guangxi, and Zhejiang. Their results showed that MMT could reduce drug abuse, the number of times the drug was injected, drug-related criminal behavior, and HIV infection.^7^ After enrolling in the MMT program, heroin users also reported improved relations with their families. The dropout rate in these target areas was 51.6% at the end of the 1-year evaluation.

Jiangsu Province is located in south-east China and its economy is relatively well-developed. Since urbanization and drug abuse are known to be associated,^8^ we considered the possibility that the effectiveness of MMT in Jiangsu Province might differ from the previous study by Pang et al^7^ which evaluated the effectiveness of MMT in poorer provinces.

In addition, it can be hypothesized that MMT is effective regardless of patient’s demographic characteristics, although few studies investigated how demographic characteristics might change the impact of MMT. Previous studies in the US reported that demographic variables did not affect the effectiveness of MMT.^9^

The objective of this study was to evaluate the impact of 1 year of MMT in the MMT clinics in Jiangsu Province, which is an economically well developed area, in terms of: 1) reduction of heroin use (total use, injection and sharing syringes), 2) increase of appropriate sexual behavior, that is, reduction of having multiple partner and increase of condom use in heterosexual intercourse, 3) reduction of antisocial behavior, 4) increase in number of people who are better off related to social and familial relationships, and 5) reduction of HIV prevalence among heroin users in MMT clinics. Among those who could follow-up, the impact of demographic variables on MMT was investigated.

## Study Design and Methods

### Setting and procedures

To help insure security, participants are forbidden to take methadone outside of the clinic. Patients must come to the clinic daily once they have enrolled in the program. The mean dosage of the therapy in the 4 clinics was 55 mg per patient. The treatment cost was 10 yuan (US$ 1.5) per day regardless of the individual dosage.

Once the patient was accepted and enrolled in the program, took part in a monthly urine test for morphine, which enabled supervision of the methadone treatment. The staff did not inform the patient informed in advance which day he/she would be tested. The test was performed using a urine test kit. Since morphine is a metabolite of heroin, the test result will be positive if the patient has taken heroin recently. This allows the patient to be supervised. Termination of the treatment did not occur if the patient provided a positive test result. Instead, a nurse or doctor would counsel the patient about the reason why he/she had used heroin while taking methadone so that related problems might be solved. The counseling provided to the patient was free of charge.

### Subjects and design

#### Research design and subjects

By the end of September 2006, a total of 1402 patients had visited the 4 MMT clinics in Jiangsu Province, China. From March to April 2006, patients were recruited and 554 patients agreed to participate in the 1st survey (i.e. these 554 patients don’t receive MMT), and from August to September 2007, 804 patients who received more than 1 year MMT participated in the 2nd survey (i.e. follow-up after 1-year MMT). Among them, 196 patients participated in both the 1st and 2nd survey. Thus, 240 patients didn’t participate in both 1st and 2nd survey. A repeated cross-sectional survey study was conducted and a questionnaire investigation was implemented. The study was conducted by researchers in Jiangsu Provincial Center for Disease Prevention and Control. All the doctors in the MMT clinics were trained to collect information from the patients. Doctors conducted face-to-face interviews with the patients in a private room. The doctors promised the patient that he/she would not be rewarded or punished regardless of whether or not he/she agreed to participate in the investigation. The doctor explained the objective of the survey and the process of participating to each patient. He then read out the information pertaining to informed consent, which was on the front page of the questionnaire. The patient ticked “Yes” if he/she agreed to participate in the investigation. The survey was anonymous, and no personal information appeared on the questionnaire. Responses were identified by unique ID numbers. The database thus created will not be shared without justifiable reason.

#### Outcome measurements

The outcome measurements were heroin use behavior, sexual intercourse behavior, antisocial behavior, social or family relationships, and HIV prevalence.

Heroin use behavior was assessed by the following 5 questions. (1) Have you ever used heroin in the last month? (2) Have you ever injected heroin in the last month? (3) How many times did you inject heroin in the last month? (4) How many times did you share syringes in the last month? (5) In the last month, how often did you contact your friends who also use heroin? The response to the first 2 questions, i.e. (1) and (2), was either yes or no. The last question, i.e. (5), was answered on a 4-point Likert scale—every day, often, occasionally, or never.

Sexual intercourse behavior was assessed by 2 questions. (1) Did you use a condom during the last sexual intercourse? (2) In the past 3 months, how many sex partners did you have? The response to the first question was either yes or no. The last question was answered using an exact number.

Antisocial behavior was assessed by 4 questions. (1) Have you ever been caught in an illegal act by the police in the last 3 months? (2) Did you steal, rob, or cheat others in the last 3 months in order to support your heroin habit? (3) In the past 3 months, did you have sex with others only to obtain money to buy drugs? (4) Have you ever sold heroin to others in the last 3 months? The response to these questions was either yes or no.

Social and family relationships were assessed by 2 questions. (1) Have you got a job at present (stable, temporary, or individual job)? (2) How do you evaluate the recent relationship between you and your family? The first question was answered as either yes or no, while the second question was answered on a 3-point Likert scale—fairly good, average, or worse.

We used different time frames for different study outcomes for heroin use. For example, we asked participants about their sexual/illegal behavior in the last 3 months rather than in only the last month because heroin use is a daily-occurring behavior while the latter is a behavior that happens less frequently. Our social and family relationships measure asks participants to evaluate their ‘recent’ relationship with family because we were interested in how the patient felt about their family in the present.

To determine the impact of MMT in preventing HIV infection, all patients were requested to undergo blood tests for the HIV antibody every year from the time of entry into the program. The only exception was patients who already had a valid HIV-positive report. Five milliliters of blood was taken from the patient by a nurse and tested by enzyme-linked immunosorbent assay in a local HIV screening laboratory. Positive blood samples were confirmed by western blot analysis in the provincial confirmative laboratory.

#### Explanatory measurements

The following explanatory variables, including demographic characteristics and socioeconomic status, were assessed: gender (male/female), age, ethnicity (Han, Zhuang, Man, or others), education (illiterate, primary school, junior middle school, senior middle school, or high school and above), marital status (unmarried, first marriage, second marriage, divorced, or widow/widower), working status (company owner, self-conducted owner, unemployed, official/clerk, worker, or others), living status (with family, with friends, alone, or other), main source of income in the last 6 months (including the money used to pay for heroin or methadone treatment (stable salary, temporary salary, supported by family or friends, or others).

#### Data analysis

Data were entered in Epidata 3.0 (Epidata Association, Odense, Denmark) and analyzed using the SPSS version 13.0 statistical software package. Continuous variables were represented as mean ± standard deviation, while categorical variables were represented as percentages. The Chi-square and Fisher’s exact tests were used to examine the differences in social and behavioral characteristics between the 2 surveys. The p value was reported for a two-tailed statistical test, while α < 0.05 was considered to be statistically significant. Multivariable logistic regression analysis was used to identify factors that affected changes in behavior related to heroin use, sexual intercourse, and social interactions. Explanatory variables were categorized on the basis of their distribution as follows: gender, 2 categories (male or female); education, 2 categories (junior middle school and below or senior middle school and above); marital status, 3 categories (never married, married (first marriage or second marriage), or divorced); working status, 2 categories (unemployed or employed); living status, 2 categories (alone or with others); and source of income, 2 categories (own salary or supported by others).

## Results

### Dropouts

By the end of September 2006, a total of 1402 patients entered the 4 clinics. There were 404 patients that dropped out during the course of the first year of treatment: among them, 143 patients were 1st survey participants. The dropout rate was 28.8% during this period. The average length of treatment among those who dropped out of this program was 22 weeks. Among those who had dropped out, 54.5% (220/404) were caught by the police for an illegal offence or crime such as continued use of heroin, robbery, or theft. Of the total number of dropouts, 27.7% (112/404) did not come to the clinic any longer for inexplicable reasons or because they broke the clinic rules. Of the total dropouts, 6.7% (27/404) left the city where they were undergoing treatment to work in another city where MMT clinics were not available.

### Demographic characteristics

The average age of patients in the 1st survey was 34.9 ± 6.2 years, while in the 2nd survey, it was 36.1 ± 6.5 years. The results of the 1st and 2nd surveys did not show any statistically significant difference in terms of gender, education, ethnicity, and living status (Table 1).

### Information on heroin use

There were 495 patients who had injected in the last month before entering the clinics and completing the 1st survey. Others used heroin by sniffing. In the 2nd survey, the percentage of heroin-use in the last month, heroin injected in the last month, and syringes shared with other heroin users in the last month were significantly lower than those in the 1st survey (heroin use, 100% vs. 17.2%; heroin injection, 89.4% vs. 14.1%; sharing syringes, 15.0% vs. 3.6%; all p <0.01).

### Sexual intercourse behavior

The percentage of patients with multiple sexual partners during the last 3 months decreased for both male and female patients in the 2nd survey in comparison to the 1st survey (male, 19.0% vs. 4.0%; female, 10.3% vs. 0.7%; p < 0.01). However, the percentage of patients using condoms during the last sexual intercourse was not statistically significant (p = 0.91).

### Antisocial behavior

The percentage caught in illegal acts by the police in the last 3 months decreased from 19.1% in the 1st survey to 3.1% in the 2nd survey (p <0.01). Illegal behavior and crimes, such as theft or robbery, or prostitution, to obtain money to buy the drug, decreased significantly in the last 3 months (all p <0.05).

### Social and family relationships

With respect to their recent relationships with their family, working status, marital status, and source of income resource were significantly improved in comparison with 1st and 2nd survey (Table 1). In addition, 82.3% of the patients in the 2nd survey felt that it had improved while only 48.0% of the patients in the 1st survey shared the same feeling (p < 0.01). Meanwhile, the probability of coming in contact with other heroin users in the last 1 month reduced from 65.2% in the 1st survey to 8.6% in the 2nd survey (p <0.01).

### HIV prevalence

There were 11 HIV-positive patients among the 1402 patients who entered the MMT clinics from March 2006 to September 2006. Two patients were confirmed after enrolment, while the other 9 were confirmed prior to enrolment. The HIV-positive rate was 0.8%. After 1 year, with the exception of 20 patients who had transferred to other newly opened clinics where they could obtain treatment conveniently, 978 patients still underwent treatment. None of the HIV-negative patients had seroconverted to the HIV-positive state.

### Number of positive urine tests

In the 1st survey, all 554 patients had positive results because they had just used heroin, while in the last month of the 2nd survey, only 92 of 804 patients (11.4%) had positive results.

### Factors affecting heroin use-related behavior

Table 2 shows the odds ratios of the variables “used heroin in the last month” and “injected heroin in the last month” according to the levels of each demographic variable. Gender, age, education, marital status, working status, living status, and source of income showed no statistically significant relationship with heroin use.

Table 3 shows the odds ratios of the variables “had more than partner in the last 3 months” and “used condom during the last sexual intercourse” according to the levels of each demographic variable. All demographic variables, with the exception of age, had no statistically significant relationship with past sexual behavior. Those who were elder were less likely to use condom in the latest sexual intercourse.

Table 4 shows the odds ratios of the variable “caught in an illegal act by the police in the last 3 months” according to the level of each demographic variable. None of the demographic variables had any statistically significant relationship with this variable.

## Discussion

The findings from the study showed that MMT contributed to a reduction of heroin use, especially in the form of injections, anti-social behaviors, and in improving social and familial relationships. Both male and female patients tended to have fewer sex partners after MMT. A longitudinal study of 196 patients showed that there was no specific demographic variable that could be attributed to promoting or deteriorating the impact of MMT. Thus, MMT treatment appeared to be effective regardless of demographic variables such as age, gender, or socioeconomic status, which is consistent with previous studies across cultures.^9^,^10^ When the blood of patients was tested after the 1-year treatment, none of them had seroconverted to HIV-positive.

Consistent with the findings of an earlier study on 8 pilot MMT clinics in China,^7^ our study also reported a reduction in HIV risk-taking behavior and an improvement in social well-being. However, the dropout rate in our study (28.8%) was lower than that in the above-mentioned study (51.6%). There were probably 3 reasons for the lower dropout rate in our study. First, the 4 MMT clinics in Jiangsu Province represent the third group in China to initiate the MMT project. Doctors and nurses employed in these 4 clinics have received training from the National Workgroup, which is now relatively well experienced. After receiving formal training, these doctors and nurses utilize their skills to deal with heroin users. Second, the 4 clinics are located in economically well-developed areas, and transportation is relatively convenient for the patients. The 8 pilot clinics that Pang studied are in relatively less affluent areas,^7^ which may affect the patient’s ability to continue treatment. Third, the average dosage in our study was 55 mg which was relatively higher than that in Pang’s research (45 mg). Many studies had reported that high retention rate was related to the relatively higher dosage.^11^–^13^

Although it is well-known that MMT is effective in reducing heroin use among addicts, a few studies have shown that demographic variables modify the effectiveness of MMT. A retrospective study conducted among female patients in methadone clinics showed that ethnicity played a role in changes in sexual behavior over the last 6 months.^14^ Thus, African-American women were less likely than Caucasion or Latina women to report changes in their sexual behavior. In our study, we found that there was no specific group in which MMT was particularly effective. The results suggest that all heroin users are recommended to receive MMT except those with medical contraindications in China. However, we could not investigate whether ethnicity was attributed to behavioral change due to lack of power, while many minorities exist in certain provinces in China. Further researches need to explore whether the current MMT operations is suitable for heroin users in minority areas in China.

The positive rate for morphine in the urine test conducted in the last month after more than 1 year of MMT treatment (11.4%) in our study was much lower than that reported in the study by Liu et al in Guizhou, China (30.0%).^15^ Petitjean et al also reported 59% urine positive rate in patients who had received a 6-week methadone maintenance treatment.^16^ Urine morphine test is a measure for supervising relapse. If it is positive, the patient would receive further consultation from the staff.

Our study showed that the percentage of patients with multiple sex partners was lower after 1-year MMT treatment, while condom use was not improved. An intervention survey conducted among MMT patients in the U.S. demonstrated that safe sex counseling and behavior intervention decreased the incidence rate of unsafe sexual behavior.^17^ Another intervention study with a control group also proved that educating patients undergoing methadone treatment could increase condom use.^18^ It can be speculated that the 4 clinics paid more attention to preventing relapse than to other negative behavior such as unsafe sex. During MMT, in addition to the daily therapy, health workers educate patients on the importance of heroin abstinence and the dangers of HIV transmission through shared syringes. In future treatment procedures, physicians and nurses should play an active role in educating patients about safe sex and encourage patients to use condoms.

After 1-year MMT, the percentage of antisocial behavior decreased substantially. A systematic review conducted by Holloway and his colleagues showed that clients in treatment program had less criminal behavior than the comparison groups.^19^ In our MMT clinics, after enrolling and participating in individual treatment, patients do not worry about the abstinence symptoms. Since heroin addiction is a costly habit, they do not need to struggle financially after they have enrolled in MMT. In several cases, the patient is more focused on his/her relationship with other family members, extent of treatment, and withdrawal from heroin dependence. The need to perform their role in the family set-up also encourages them to find a job. The more concerned they are about family relationships, the greater is the possibility that their relationships would improve.

Several studies conducted in different countries have proved that MMT is effective in treating heroin addiction.^20^–^22^ In recent years, it has attracted more attention since it is linked with reduction of HIV transmission.^23^ The results have shown that MMT is effective in China in both undeveloped^7^ and developed areas (our study). This indicates that the current MMT operations in China are suitable for the Chinese conditions. In both studies, the percentage of patients injecting heroin and exhibiting antisocial behavior decreased after the 1-year treatment, while the dropout rate was lower in our study. A recent study in Hong Kong showed that the location of methadone clinics was as important as their number.^24^ When setting up more MMT clinics, the location of the clinics should be convenient for heroin users to help cover a larger target population.

It was shown that the effectiveness of MMT depended on the quality of the treatment in terms of factors such as the methadone medication dose, support from society, and financial stability to maintain access to the MMT clinic.^25^–^29^ Thus, the extent to which heroin use is reduced, which benefits both the individual patient and the society, would depend on the quality of the treatment. Psychological consultation and support on preventing relapse, obtaining vocational training, finding a new job, and forming a harmonious relationship with the general community were constantly provided to the patients by the doctors and nurses in the clinic; this may have contributed to the success of the treatment.^29^–^31^

One limitation of our study is that not all of the samples in the second survey were investigated in the first survey. In other words, our longitudinal study had a selection bias. However, the distribution of demographic variables between those that could be followed and those that could not be followed did not differ significantly in our sensitivity analysis (data not shown). In addition, some of the patients who were investigated in the first survey dropped out over time. It may be speculated that the percentage reduction in negative behavior observed in this study might be an overestimation.

Another limitation of our study is that there is no parallel control group when the efficacy of MMT is evaluated. Ethical considerations restrict the recruitment of community heroin users if MMT is not offered to them. The mass media in each city publicized the benefits of MMT when the local MMT clinics began operations, and the MMT clinics sent brochures to patients and urged them to bring their friends who also used heroin to the clinic. It was difficult to contact heroin users in the community if they were reluctant to come to the clinic. The findings of our study showed that the percentage of behavior change was significant and substantial and was considered most likely due to MMT. However, it would be desirable to compare the behavioral changes among heroin users who received MMT with those who did not receive MMT. One possible method would be to compare the HIV-related behavior of heroin users in Jiangsu Province with that of heroin users in other cities that do not have MMT clinics. Further studies with a control group are required to show the effectiveness of MMT in reducing risky behavior related to HIV transmission.

## Figures and Tables

**Table 1:** Comparison of the demographic characters in the first and second surveys.

Variables	First survey		Second survey		P
			
	n	Proportion (%)	n	Proportion (%)	
**Gender**					
Male	434	78.3	614	76.4	NS
Female	120	21.7	190	23.6	
**Education**					
Illiterate/primary school	35	6.3	60	7.5	NS
Junior middle school	342	61.7	463	57.6	
Senior middle school	167	30.2	264	32.8	
High school and above	10	1.8	17	2.1	
**Working status**					
Unemployed	415	74.9	504	62.7	<0.01
Employed	139	25.1	300	37.3	
**Ethnicity**					
Han	537	96.9	783	97.4	NS
Others	17	3.1	21	2.6	
**Marital status**					
Unmarried	271	48.9	308	38.3	<0.01
Married	185	33.4	369	45.9	
Divorced/ Widow/ Widower	98	17.7	127	15.8	
**Living status**					
With family/friends	484	87.4	694	86.3	NS
Live alone	70	12.6	110	13.7	
**Source of income resource**					
Stable/temporary salary	97	17.5	288	35.8	<0.01
From family/friends	321	57.9	368	45.8	
From social welfare/others	136	24.6	148	18.4	

**Table 2:** Multivariate logistic regression analysis of demographic variables on heroin use during the last month.

Demographic variables	Percentage (N = 196)	Used heroin	Injected heroin
			
		OR (95% CI)	OR (95% CI)
**Gender**			
Male	80.6	1.00	1.00
Female	19.4	0.89 (0.36–2.21)	1.07 (0.42–2.68)
**Age**		1.00 (0.94–1.06)	0.97 (0.91–1.04)
**Education**			
Junior middle school and below	64.3	1.00	1.00
Senior middle school and above	35.7	0.80 (0.38–1.69)	0.79 (0.36–1.75)
**Marital status**			
Never married	45.9	1.00	1.00
Married	36.3	1.39 (0.59–3.27)	1.63 (0.66–4.05)
Divorced	17.8	1.78 (0.62–5.09)	1.72 (0.55–5.40)
**Working status**			
Unemployed	73.5	1.00	1.00
Employed	26.5	1.22 (0.46–3.27)	0.79 (0.26–2.40)
**Living status**			
Live alone	13.3	1.00	1.00
With family/friends	86.7	2.28 (0.61–8.55)	2.52 (0.54–11.75)
**Source of income**			
Own salary	17.3	1.00	1.00
From family/friends	82.7	1.13 (0.35–3.70)	1.11 (0.30–4.15)

**Table 3:** Multivariate logistic regression analysis of demographic variables on past sexual behavior.

Demographic variables	Percentage (N = 120)	Had more than 1 partner in the last 3 months	Used condom during the last sexual intercourse
			
		OR (95% CI)	OR (95% CI)
**Gender**			
Male	77.5	1.00	1.00
Female	22.5	N/A	0.50 (0.18–1.34)
**Age**		1.00 (0.83–1.20)	0.91 (0.84–0.98)
**Education**			
Junior middle school and below	67.5	1.00	1.00
Senior middle school and above	32.5	0.87 (0.08–9.35)	1.37 (0.60–3.14)
**Marital status**			
Never married	40.8	1.00	1.00
Married	43.4	0.40 (0.03–5.96)	2.22 (0.83–5.91)
Divorced	15.8	2.04 (0.12–34.68)	2.07 (0.58–7.35)
**Working status**			
Unemployed	75.8	1.00	1.00
Employed	24.2	0.52 (0.01–25.64)	0.44 (0.10–1.95)
**Living status**			
Alone	10.0	1.00	1.00
With family/friends	90.0	N/A	0.62 (0.16–2.34)
**Source of income**			
Own salary	18.3	1.00	1.00
From family/friends	81.7	0.3 (0.01–15.95)	0.29 (0.06–1.38)

**Table 4:** Multivariate logistic regression analysis of demographic variables on antisocial behavior in the last 3 months.

Demographic variables	Percentage (N = 196)	Caught by the police
		
		OR (95% CI)
**Gender**		
Male	80.6	1.00
Female	19.4	1.79 (0.41–7.84)
**Age**		1.07 (0.97–1.19)
**Education**		
Junior middle school and below	64.3	1.00
Senior middle school and above	35.7	0.42 (0.08–2.06)
**Marital status**		
Never married	45.9	1.00
Married	36.3	1.29 (0.27–6.08)
Divorced	17.8	0.35 (0.03–3.76)
**Working status**		
Unemployed	73.5	1.00
Employed	26.5	0.33 (0.02–4.42)
**Living status**		
Live alone	13.3	1.00
With family/friends	86.7	0.71 (0.12–4.25)
**Source of income**		
Own salary	17.3	1.00
From family/friends	82.7	0.39 (0.03–5.00)
